# Secreted therapeutics: monitoring durability of microRNA-based gene therapies in the central nervous system

**DOI:** 10.1093/braincomms/fcab054

**Published:** 2021-04-01

**Authors:** Marina Sogorb-Gonzalez, Carlos Vendrell-Tornero, Jolanda Snapper, Anouk Stam, Sonay Keskin, Jana Miniarikova, Elisabeth A Spronck, Martin de Haan, Rienk Nieuwland, Pavlina Konstantinova, Sander J van Deventer1, Melvin M Evers, Astrid Vallès

**Affiliations:** 1 Department of Research & Development, uniQure Biopharma N.V, ., Amsterdam, The Netherlands; 2 Department of Gastroenterology and Hepatology, Leiden University Medical Center, Leiden, The Netherlands; 3 Laboratory of Experimental Clinical Chemistry, Amsterdam UMC, University of Amsterdam, Amsterdam, The Netherlands, and Vesicles Observation Center, Amsterdam UMC, University of Amsterdam, Amsterdam, The Netherlands

**Keywords:** Gene therapy, microRNA, extracellular vesicles, huntington disease, spinocerebellar ataxia

## Abstract

The preclinical development of microRNA-based gene therapies for inherited neurodegenerative diseases is accompanied by translational challenges. Due to the inaccessibility of the brain to periodically evaluate therapy effects, accessible and reliable biomarkers indicative of dosing, durability and therapeutic efficacy in the central nervous system are very much needed. This is particularly important for viral vector-based gene therapies, in which a one-time administration results in long-term expression of active therapeutic molecules in the brain. Recently, extracellular vesicles have been identified as carriers of RNA species, including microRNAs, and proteins in all biological fluids, whilst becoming potential sources of biomarkers for diagnosis. In this study, we investigated the secretion and potential use of circulating miRNAs associated with extracellular vesicles as suitable sources to monitor the expression and durability of gene therapies in the brain. Neuronal cells derived from induced pluripotent stem cells were treated with adeno-associated viral vector serotype 5 carrying an engineered microRNA targeting *huntingtin* or *ataxin3* gene sequences, the diseases-causing genes of Huntington disease and spinocerebellar ataxia type 3, respectively. After treatment, the secretion of mature engineered microRNA molecules was confirmed, with extracellular microRNA levels correlating with viral dose and cellular microRNA expression in neurons. We further investigated the detection of engineered microRNAs over time in the CSF of non-human primates after a single intrastriatal injection of adeno-associated viral vector serotype 5 carrying a *huntingtin*-targeting engineered microRNA. Quantifiable engineered microRNA levels enriched in extracellular vesicles were detected in the CSF up to two years after brain infusion. Altogether, these results confirm the long-term expression of adeno-associated viral vector serotype 5-delivered microRNAs and support the use of extracellular vesicle-associated microRNAs as novel translational pharmacokinetic markers in ongoing clinical trials of gene therapies for neurodegenerative diseases.

## Keywords:

Gene therapy, microRNA, extracellular vesicles, Huntington disease, spinocerebellar ataxia

**Abbreviation list**

AAV = adeno-associated virus

AAV5-miATXN3 = adeno-associated virus serotype 5 encoding an engineered microRNA targeting *ATXN3* gene sequence

AAV5-miHTT = adeno-associated virus serotype 5 encoding an engineered microRNA targeting *HTT* gene sequence

Ago2 = Argonaute protein type2

ATXN3 = ataxin 3

CAGp= cytomegalovirus early enhancer element fused to chicken b-actin promoter cDNA = complementary DNA

CED = convention-enhanced delivery

DPBS = Dulbecco's phosphate-buffered saline

EVs = extracellular vesicles

FDA = Food and Drug administration gc = genome copies

GFAP = glial fibrillary acidic protein

GFP = green fluorescence protein

HD = Huntington disease

HDL = high density lipoprotein

HTT = huntingtin iPSCs = induced pluripotent stem cells

MAP2 = microtubule-associated protein 2

miRNA = microRNA

MOI = multiplicity of infection

MRPS = Microfluidic resistive pulse sensing polyQ = polyglutamine pre-miRNA = precursor miRNA

RI = refraction index

RISC = RNAi silencing complex

RNAi = RNA interference

RT-PCR = reverse transcription polymerase chain reaction

SCA3 = spinocerebellar ataxia type 3

SEC = size exclusion chromatography

SEM = Standard error of the mean

TEM = transmission electron microscopy https://mc.manuscriptcentral.com/braincom

## Introduction

The identification of causal mutations in many neurodegenerative diseases has opened the door to the development of novel disease-modifying therapies. In the case of polyglutamine (polyQ) diseases, caused by inheritance of CAG triplet-repeat expansions, gene silencing approaches are being investigated to lower the presence of toxic polyQ proteins.[1–5] Next to the use of antisense oligonucleotides, one of the most advanced lowering strategies is microRNA (miRNA)-based gene therapy.[6] This approach is based on the design of RNA interference (RNAi) molecules complementary to the mutation-carrying mRNA and inserted into a precursor miRNA (pre-miRNA) backbone. Engineered pre-miRNAs, likewise their endogenous counterparts, are processed into mature ~21 nucleotide miRNA molecules which are incorporated in the RNAi silencing complex (RISC). Activated miRNA-RISC complex binds to target mRNA based on sequence complementarity resulting in translational repression or enzymatic cleavage.[7] Engineered miRNA-based treatments rely on the administration of an adeno-associated viral (AAV) vector containing an expression cassette of the therapeutic miRNA precursor. The episomal persistence of the viral expression cassette results in a continuous production of the therapeutic agent and long-term gene suppression after one-time administration, bypassing the need of re-administration. Proof-of-concept studies have demonstrated that AAV-delivered miRNAs can be used for safe, effective and durable lowering of toxic mutant proteins in polyQ-related neurodegenerative disorders.[3,8–10] For Huntington disease (HD), the most common polyQ disorder, infusion of a serotype 5 AAV-delivered miRNA targeting the huntingtin gene (*HTT*) sequence (AAV5-miHTT) resulted in efficient and well-tolerated HTT protein lowering in the brain of different HD animal models.[8,11–13] Long-term HTT lowering was associated with prevention of neuronal dysfunction and functional improvement in HD mouse models, supporting the Food and Drug Administration (FDA) approval and initiation of a Phase I/II clinical trial in early manifest HD (Clinicaltrials.gov, NCT04120493).

The translation of miRNA-based gene therapies to the clinic is faced with meaningful challenges.[5,14] One of the main obstacles for neurodegenerative diseases is the relative inaccessibility of the CNS. Different routes of administration have been investigated to achieve a wide distribution of viral particles and sufficient expression of engineered miRNA molecules in affected brain regions.[15] Studies in non-human primates show that AAV5 infusion in the striatum leads to efficient transduction not only in the primary target structures, but also in cortical projection areas, which become also affected in HD.[16,17] Besides delivery, there is an urgent need to establish accessible biomarkers which are indicative of dosing, safety and efficacy in patients after treatment. This is especially important for AAV-based gene therapy approaches, where a one-time administration results in long-term expression of active therapeutic molecules. Finding reliable translational measurements to monitor vector expression and levels of therapeutic molecules would be an important step towards establishing pharmacokinetic measures to predict efficacy and long-term durability of miRNA-based gene therapies in ongoing and future clinical studies.

Recent evidence has shown that signaling molecules such as proteins, lipids and RNAs can be released from the cells of origin within extracellular vesicles (EVs).[18,19] The term "EVs" includes exosomes, microvesicles and other small vesicles that are released into the extracellular environment by fusion of multivesicular bodies or by direct budding of the plasma membrane. Since they are found in all body fluids, EV profiles and cargos are emerging as clinically useful and non-invasive prognostic biomarker carriers in cancer and brain disorders.[20,21] In the case of neurodegenerative disorders, the trafficking of molecules from the inaccessible CNS to CSF mediated by EVs allows the detection of pathology-related molecules, which remain protected from proteases and nucleases present in the liquid. Numerous studies have reported altered CSF miRNA expression profiles for Alzheimer disease, Parkinson disease and multiple sclerosis.[22–24] However, in order to establish reliable biomarkers, it is important to understand that EV loading is not a random process and cellular overexpression does not always imply packaging into EVs. For instance, a recent study identified a set of endogenous miRNAs preferentially released within EVs across different cell types.[25] Among those, miR-451a was found to be the most highly EV-enriched miRNAs relative to the cellular levels. Therefore, characterization of preferentially exported molecules and correlation with cellular status is highly relevant.

In this study, we evaluated the secretion of engineered therapeutic miRNAs from neuronal cells via EVs after AAV5 delivery. We further investigated the potential use of extracellular miRNAs in biological fluids as suitable sources for measurements to monitor the long-term durability of active miRNA molecules in the brain after one-time AAV5 administration. For this purpose, we selected two therapeutic miRNAs designed to target huntingtin gene (miHTT) and ataxin 3 gene (miATXN3), the disease-causing genes of HD and spinocerebellar ataxia type 3 (SCA3), respectively , both delivered via AAV5 (AAV5-miHTT and AAV5-miATXN3).[8,10] Our results show that AAV5-delivered therapeutic miRNA molecules are released from neuronal cells in a dose-dependent manner in association with EVs and protein complexes. Furthermore, a reliable detection of therapeutic miRNA in biological fluids in non-human primates up to two years after dosing confirms the translational value of extracellular miRNAs to monitor the expression and durability of therapeutic miRNAs directly delivered in the brain. The present findings have important implications for the long-term monitoring of ongoing and future clinical studies of miRNA-based gene therapies for CNS disorders.

**Material and methods**

## Differentiation of forebrain neuronal cultures from human induced pluripotent stem cells (iPSCs)

We selected human iPS cells from a HD patient containing 71 CAG repeats (ND42229*B, Coriell Institute Stem Biobank, New Jersey, USA). These cells were generated from human HD fibroblasts (GM04281, Coriell Institute Stem Biobank) and reprogrammed with six factors (OCT4, SOX2, KLF4, LMYC, LIN28, shRNA to P53) using episomal vectors. iPSCs were maintained on matrigel coating with mTeSR medium (StemCell Technologies, Vancouver, Canada) for several passages, following the manufacturer's instructions. Karyotype analysis was performed to confirm chromosomal stability before differentiation. Non-differentiated colonies were released using ReLeSR reagent during each passage and split 1:5-20 (StemCell Technologies). For the neural induction, cells were plated onto AggreWellTM 800 plate at day 0 as $3x10^6$ cells per well in STEMdiff™ Neural Induction Medium (StemCell Technologies). At day 5, embryoid bodies were formed and replated onto poly-D-lysine/laminin coated 6-well plates. Coating was prepared with poly-D-lysine hydrobromide (0,1 mg/mL) and Laminin from Engelbreth-Holm-Swarm murine (0,1 mg/ml) (Sigma-Aldrich). At day 12, the neuronal rosettes were selected using STEMdiff™ Neural Rosette Selection Reagent (StemCell Technologies) and replated in poly-D-lysine/laminin coated plates. The following day, differentiation of neural progenitor cells was initiated using STEMdiff™ Neuron Differentiation Kit (StemCell Technologies). From day 19, cells were matured using STEMdiff™ Neuron Maturation Kit for a minimum of two weeks (StemCell Technologies). Cell cultures were daily monitored during the whole process.

## Vector design and production

The AAV5 vector encoding cDNA of the miHTT cassette was produced using a baculovirus-based AAV production system (uniQure, Amsterdam, the Netherlands) as described previously.[8] Expression was driven by a combination of the cytomegalovirus early enhancer element and chicken b-actin promoter (CAGp), and the transcription unit was flanked by two non-coding AAV-derived inverted terminal repeats.

## Transduction iPSC-derived neuronal cultures

Two days after seeding in poly-D-lysine/laminin coated plates ($1x10^5$ cells), neuronal cells were transduced with three doses of AAV5-miHTT (multiplicity of infection (MOI) $1x10^5$, $1x10^6$ and $1x10^7$ viral particles per cell, respectively) or two doses of AAV5-miATXN3 (MOI $1x10^6$ and $1x10^7$ viral particles per cell).

## Intrastriatal injection in non-human primates and CSF collection

This study in non-human primates was performed by Covance Preclinical Services in Munster (Germany) in compliance with the German Animal Welfare Act and approved by the local Institutional Animal Care and Use Committee. This study was ethically approved by the Niedersächsisches Landesamt für Verbraucherschutz und Lebensmittelsicherheit (LAVES, permit number AZ 33.19-42502-04-16/2363; Oldenburg, Germany) and the institutional Tierschutzausschuss (IACUC), prior to the initiation of the *in life* phase. Cynomolgus monkeys (*Macaca fascicularis*) aged 2.6-3.5 years, were randomized into two cohorts and kept in live for either 6 months (Cohort 1) or 24 months (Cohort 2). Animals in each cohort were injected with formulation buffer or AAV5-miHTT locally in the caudate and putamen (100 µl/region) by using MRI-guided convention-enhanced delivery (CED). Animals in cohort 1 were separated in two groups (each group included three males and three females) and received low dose $2x10^{12}$ gc/brain or high dose $2x10^{13}$ gc/brain of AAV5-miHTT. Animals in cohort 2 received high dose $2x10^{13}$ gc/brain of AAV5-miHTT. Several longitudinal CSF samples were taken, namely on pre-dose (day 0), month 1, 3, 4,5 and 6 (cohort 1) and 12, 15, 18, 21 and 24 (cohort 2). For CSF sample collection, animals were anesthetized with ketamine (10 mg/kg) and dexmedetomidine (0.015 mg/kg). A pencil-point needle for pediatric use was used for CSF withdrawal at level L3 - L6 and the withdrawn volume was substituted with the same volume of artificial CSF. CSF samples (approximately 1 mL) were collected at each time point, centrifuged for 10 min at 2000 x g, 4°C, divided into 2 aliquots (0.5 mL each), snap frozen and stored at - 80°C until further analysis.

## Necropsy procedures and tissue collection

Cynomolgus monkeys were sacrificed at 6 months for cohort 1 and 24 months for cohort 2 after AAV5-miHTT administration by sedation with intramuscular injection of ketamine hydrochloride. The brains were carefully removed and coronally sliced into 3mm slices. In total 80 punches were taken from 3mm brains slices across different structures of interest.

## RNA/DNA isolation and real-time qPCR

RNA was isolated from neuronal cells and non-human primate brain tissue with Direct-zol™ according to the manufacturer's protocol (Zymo Research; CA, USA). To determine miRNA expression levels, two-step RT-qPCR was performed TaqMan Fast Universal kit (Thermo Scientific, MA, USA), and custom stem-loop primer/probe for detection of miHTT and miATXN3 (Thermo Scientific). Complementary DNA (cDNA) was diluted two-fold in nuclease-free water, of which 4 μL per reaction were analyzed by TaqMan qPCR. Expression levels of miHTT and miATXN3 were calculated based on a standard line with synthetic RNA oligos (Integrated DNA Technologies, IA, USA). Expression levels of endogenous miRNAs were measured as internal control, using primer/probe U6 snRNA (001973), hsa-miR-16 (000391), has-miR-21-5p (000397), and cel-miR-39 (000200) from ThermoFisher Scientific.

For viral DNA isolation, neuronal cultures were processed using DNeasy Blood & Tissue Kit (Qiagen, CA, USA) following the manufacturer's protocol. AAV5 vector genome copies were measured by qPCR using SYBR Green (Applied Biosystems, CA, USA) and specific primers against CAG promoter (5'-3' primer sequences: Forward GAGCCGCAGCCATTGC, reverse CACAGATTTGGGACAAAGGAAGT). A standard line with expression plasmids was used to calculate the genome copies per microgram of genomic DNA (gDNA).

## Isolation of EVs from culture media by precipitation

Medium from transduced neuronal cultures was refreshed every two days, collected on day 5 and day 12 after transduction and centrifuged at 4,000 x g for 15 minutes to remove cells and cell debris. EVs were isolated with a polymer-based precipitation buffer (ExoQuick-TC, System Bioscience, California, USA) according to the manufacturer's protocol. Briefly, 3 mL of ExoQuick buffer was added to 10 mL of conditioned medium, gently mixed, and incubated at 4°C overnight. Next day, EVs were collected at 1,500 x g for 30 minutes and the supernatant was discarded. The residual solution was additionally centrifuged at 1,500 x g for 10 minutes.

EV pellets were resuspended in appropriate buffers and stored at -80°C for further experiments. For miRNA detection, EV pellets were resuspended in 300 µL TRIzol (Invitrogen) and RNA content was isolated by using Direct-zol™ (Zymo Research; CA, USA), according to the manufacturer's protocol.

## Isolation of EVs from culture media by size-exclusion chromatography

Medium from neuronal cultures was collected and centrifuged at 4,000 x g for 15 minutes to remove cell and cell debris. Separation of EVs from protein complexes (including HDL) was achieved by SEC with qEV10 columns (Izon Science; New Zealand). Briefly, after washing the column with PBS, 10 mL of medium was loaded on the column and 26 fractions of 5mL were collected. Every fraction was concentrated to 300 µL by Amicon® Ultra-15 Centrifugal Filter Units (10 kDa molecular weight cut-off) by centrifugation at 4,000 x g for 15 minutes at 4 °C. For miRNA detection, 300 µL TRIzol (Invitrogen) were added to each fraction and RNA content was isolated by using Direct-zol™ (Zymo Research; CA, USA), according to the manufacturer's protocol.

## Isolation of EV-associated miRNA from CSF

RNA was isolated from a total of 500 µL CSF, using the exoRNeasy Serum/Plasma kit (Qiagen), as per protocol description, enriching for RNA from EVs. The sample was spiked in with miR-39, in order to control for possible variations in RNA isolation efficiency. Subsequently, TaqMan-based RT-PCR was carried out with custom stem-loop primers and probes for detection of miHTT and cel-miR-39, using 8µL as RNA template. The cDNA was diluted to a final volume of 35µL (15µL cDNA + 20µL RNAse free water). Results were expressed as miHTT molecules/mL CSF, corrected for variations in miR-39 spiked in the RNA samples.

## Western blotting

EV precipitates were lysed using 100 µL radioimmunoprecipitation assay (RIPA) buffer (Sigma-Aldrich, St. Louis, MO, USA) supplemented with protein inhibitor cocktail (cOmplete™ ULTRA Tablet; Roche, Basel, Switzerland). Total protein concentration was quantified using a Bradford Protein Assay (Bio-Rad, Hercules, CA, USA) and absorbance was measured at 600 nm on the GloMax Discover System (Promega, Wisconsin, USA). Equal amounts of sample protein (20 µg) were incubated with β-mercaptoethanol-containing Laemmli buffer at 95 °C for 5 minutes. Proteins were separated using 4-20% Mini-Protean TGX Stain-Free Protein Gel (Bio-Rad). Samples were transferred to polyvinylidene difluoride (PVDF) membranes by Trans-Blot Turbo Transfer system (Bio-Rad) using the “Mixed molecular-weight” protocol (5-150 kDa) at 1.3A, up to 25V for 7 minutes. Blots were incubated with 3% Blotting-Grade blocker (Bio-Rad) in Tris Buffered Saline (TBS) for 1 hour at room temperature, followed by immunoblotting with the selected primary antibody overnight at 4°C (Table 1). Chromogenic signals were detected after 2 hours incubation with HRP-conjugated secondary antibodies (Table 1) and 5 min incubation with SuperSignal Pico sensitivity Substrate (Thermo Scientific) using ChemiDoc Touch Gel Imaging System (Bio-Rad).

**Table 1: List of primary and secondary antibodies.**

| | Antibodies | Company | Reference | Concentration |
|---|---|---|---|---|
| EV/exosomes | **CD63** | System Biosciences | EXOAB-CD63A-1 | 1:1000 |
| | **Alix** | Abcam | Ab76608 | 1:1000 |
| | **TSG101** | Abcam | Ab30871 | 1:1000 |
| EV/microvesicles | **Calnexin** | Abcam | Ab92573 | 1:20000 |
| Lipoproteins | **ApoE** | Abcam | Ab58475 | 1:1000 |
| RISC complex | **Ago2** | EMD Millipore | 07-590-25UG | 1:1000 |
| Cytoplasm | **α-tubulin** | Abcam | Ab7291 | 1:1000 |
| Secondary HRP antibody | **HRP goat anti-rabbit** | Abcam | Ab97051 | 1:20000 |
| | **HRP rabbit anti-mouse** | Dako | P0260 | 1:20000 |

## Flow cytometry

Samples were analyzed by APOGEE flow cytometer (Hemel Hempstead, Hertfordshire, UK). EV-containing samples (5 μL) were incubated with 5 μL of a solution containing a monoclonal antibody (CD63 or CD81) or lactadherin and diluted with PBS to a final volume of 50 μL. These samples were incubated in the dark for 15 minutes at room temperature before the addition of 300 μL PBS. Samples were analyzed for 1 minute by FCM using a FACSCalibur with CellQuest software (BD) at a flow rate of 60 μL/minute. The detector settings used throughout the experiments and the calculation of EV concentration were as described previously.[26]

https://mc.manuscriptcentral.com/braincom

## Microfluidic resistive pulse sensing (MRPS)

MRPS (nCS1, Spectradyne LLC, Torrance CA, USA) was used to measure particle size and concentration of all samples. MRPS applies the Coulter Principle, relating the change in electrical impedance of a particle passing through a nanoconstriction to the volume of the particle traversing the nanoparticle tracking analyzer constriction to determine particle size and concentration. All samples were diluted 2-fold or 5-fold in 100 kDa-filtered (Vivaspin) 0.1 % (w/v) bovine serum albumin in Dulbecco's phosphate-buffered saline (DPBS). All samples were measured with a TS-400 cartridge at 4 V. To ensure the particle size distributions were representative of the sample, a minimum of 1,000 particles were counted. Filters were applied based on the recommendations of the manufacturer for the TS-400 cartridge before any data were analyzed to exclude false-positive signals.

## Transmission electron microscopy (TEM)

EVs were fixed at room temperature overnight by 0.1% (weight/volume, w/v) paraformaldehyde (Electron Microscopy Sciences, Hatfield, PA). Next, a 200-mesh EM copper grid with formvar coating (Electron Microscopy Sciences) was put on top of a sample (10 μL) and incubated for 7 minutes at room temperature. The grids were transferred to 1.75% uranyl acetate (w/v) for negative staining. The grid was imaged using a Tecnai 12 TEM (FEI Company, Eindhoven, The Netherlands), operated at 80 kV. Blind analysis and caption of representative pictures was performed at the Electron Microscopy Centre Amsterdam (EMCA), at Amsterdam Medical Centre (Amsterdam, The Netherlands).

## Statistics

Mean values were used for statistical analyses. Data are expressed as means ± SEM. Relationship between viral dose, transgene cellular expression and levels of extracellular EV-associated miRNAs were examined using linear regression analysis. The level of statistical significance was set at $p < 0.05$.

## Data and materials availability

Data associated with this study are available in the main text or the supplementary materials. AAV5-miHTT and AAV5-miATXN3 are proprietary to uniQure and are not available without at least an MTA.

## Results

### Engineered miRNA-based therapeutic candidates for inherited neurodegenerative diseases

In order to validate our hypothesis that engineered miRNAs delivered in the CNS can be detected extracellularly in biological fluids, we first evaluated their secretion from neuronal cells after AAV5-treatment. For this purpose, a neuronal system based on iPSCs from an HD patient was selected. iPSCs were differentiated into neuronal progenitor cells and matured into forebrain-like neuronal cells which were positive for microtubule-associated protein 2 (MAP2) neuronal marker. Glial cells positive for glial fibrillary acidic protein (GFAP) astrocytic marker were also found to a lesser extent (Supplementary Figure 1). We designed two miRNA sequences, both inserted in the pre-miR-451a backbone[27] (Fig. 1A) and incorporated into an AAV5 capsid. One miRNA candidate was designed to target the *HTT* sequence (miHTT) as a potential treatment for HD.[8] The other miRNA candidate targets ataxin-3 (*ATXN3*) sequence (miATXN3), the disease-causing gene of SCA3.[10] After nuclear expression by RNA polymerase II, engineered miRNAs are processed from primary transcripts to hairpin-containing precursors (pre-miRNA). Unlike other miRNAs, pre-miR-451 stem-loop structure is too short to be cleaved by the endoribonuclease Dicer and it is directly processed by protein argonaute-2 (Ago2) into mature miRNA molecules. Mature miRNAs are incorporated in the RISC complex and together bind the target mRNA based on sequence complementarity.[28] The processing of pre-miR-451 engineered miHTT and miATXN3 constructs into mature molecules has been previously determined by RNA sequencing in human and murine models.[10,29] Moreover, efficacy of both AAV5-miHTT and AAV5-miATXN3 treatment to suppress target genes has been demonstrated in human neuronal cells, brain organoids and animal models achieving successful lowering of HTT and ATXN3 protein, respectively.[10–12,30]

### AAV5-delivered therapeutic miRNAs are secreted from human iPSC-derived neurons

Neuronal cells were transduced with three increasing doses of AAV5-miHTT multiplicity of infection (MOI) 1x$10^5$, 1x$10^6$ and 1x$10^7$ viral particles per cell) or two doses of AAV5-miATXN3 (MOI 1x$10^6$ and 1x$10^7$ viral particles per cell). Successful transduction and transgene expression were confirmed by green fluorescent protein (GFP) five days after AAV5-GFP treatment (Supplementary Figure 1). Dose-dependent genome copies (gc) were detected for AAV5-miHTT and AAV5-miATXN3 treatment, with the highest dose generating approximately 1x$10^8$ gc/µg of input gDNA (n=3; Fig. 2A and B). To quantify the expression levels of engineered miRNAs after AAV5 treatment, we used a method specific for the detection of active mature miRNA (guide strand) based on TaqMan reverse transcription (RT)-quantitative (q)PCR. As expected, higher levels of mature miHTT and miATXN3 molecules were measured in neuronal cells at increasing doses of AAV5 (n=3) (Fig. 2C and 2D).

It has been recently established that miRNAs are not exclusively cellular but can be secreted via EVs into peripheral fluids or cell-culture media.[18] We first investigated the secretion of pre-miR-451 engineered miRNAs by enriching EVs from the culture media after AAV5 treatment. Due to large culture media volumes and the expected low RNA abundance, a precipitation-based method was used for EV isolation. Total RNA was isolated from the EV-enriched pellet and mature miRNA molecules were quantified by TaqMan qPCR. Increasing levels of extracellular miHTT and miATXN3 molecules were detected in association with EVs secreted from neuronal cells at increasing AAV5 doses (Fig. 2E and 2F). Stable levels, up to approximately 1x$10^5$ miRNA molecules/ml medium were found at 5 and 12 days after one-time AAV5 treatment. In all experiments, we detected comparable levels of endogenous miR-16 miRNA, independent of the dose and time point, confirming the replicable isolation of extracellular miRNAs by this method (Supplementary Figure 2). To validate these results, independent experiments with other doses of AAV5-miHTT were performed (MOI 6.7x$10^5$, 6.7x$10^6$, 3.33x$10^7$ viral particles per cell). As previously reported, we observed a steady correlation between AAV5-dose (log10) and relative cellular miHTT expression ($R^2$=0.9275, p=0.002) (Fig. 2G), confirming a dose-dependent transduction and transgene expression in iPSC-derived neurons. Similarly, a strong correlation was observed between AAV5-dose and extracellular miHTT molecules ($R^2$=0.8766, p=0.006) (Fig. 2H), and between cellular transgene expression and extracellular miHTT molecules ($R^2$=0.9431, p=0.001) (Fig. 2I). Since we used a method specific for the active mature miRNA (guide strand), extracellular miRNA content is the result of cellular transgene expression, processing and secretion of AAV5-delivered miRNA. This confirms that pre-miR-451-derived engineered miRNAs are stably secreted by neuronal cells after AAV5 transgene expression.

## Isolation of EVs by precipitation results in co-isolation of other miRNA-bound particles

Precipitation-based isolation of EVs, such as ultracentrifuge or precipitating agents, are among the most widely applied techniques for vesicle isolation. These methods have the advantage to achieve high yield of RNA, yet possibly attributable to co-isolation of RNA-bound proteins including lipoproteins and silencing protein complexes.[31] To investigate the population of particles isolated from cultured media by precipitation, we assessed the presence, size, and morphology of vesicular and non-vesicular species by several methods. Western blot analyses confirmed the detection of exosomal markers (CD63, Alix and TSG-101), microvesicles (calnexin), and RISC complex (Ago2) in EV pellets precipitated from conditioned culture media (Fig. 3A). In contrast, EV pellets were negative for intracellular marker α-tubulin, confirming the absence or low abundance of apoptotic cell bodies in the samples (Fig. 3A). Distribution of particles based on size was measured by microfluidic resistive pulse sensing. The highest concentration of particles ranged between 60-80 nm diameter, characteristic of EV populations (Fig. 3B). Flow cytometry analysis confirmed the presence of vesicles exposing CD63 and CD81, tetraspanins classically used as specific exosomal markers. Moreover, vesicles were also positive for lactadherin, a protein binding to phosphatidylserine, considered one of the best current markers for most EVs[32] (Supplementary Figure 2). Particles with a refraction index (RI) < 1.42 were characterized as EVs, and discriminated from lipoproteins.[33] Isotype $IgG_1$ labeled samples were used as controls and showed low or undetectable levels, confirming the specificity of the signal measured by EV-marker antibodies (Supplementary Figure 2). The morphology of the vesicle population was assessed by TEM. Vesicle structures in a range size of 100-150 nm were visible in samples from control cells (incubated with formulation buffer) and AAV5-miATXN3 transduced cells, but not in unconditioned culture media (Fig. 3C and Supplementary Figure 2). Notably, a significant amount of smaller protein complexes was observed in all three conditions, suggesting the presence of soluble proteins in these samples. Taken together, these results show the successful enrichment of EVs by precipitation. However, co-isolation of other protein complexes, likely associated with miRNAs, might influence the detection levels of extracellular miRNAs. To investigate the nature of extracellular miRNAs, we analyzed the effect of RNase A and detergent treatment on the miRNA levels in EV pellets precipitated from neuronal cells. Treatment of EV pellets with RNase A reduced the relative expression levels by 87% for therapeutic miATXN3 and endogenous let-7a levels, yet completely degraded endogenous levels of miR-16 and spike-in cel-miR-39 (Fig. 3D). However, lysis of vesicles with detergent (Triton X-100) prior to RNase A resulted in complete degradation of miATXN3 and let-7a (Fig. 3D). These results indicated that when EVs are isolated by precipitation-based methods, extracellular miATXN3 and endogenous let-7a molecules are only partially present and protected within EVs, and that non-vesicular miRNAs highly contributed to the extracellular levels of miRNA.

## Engineered miRNAs are secreted from neuronal cells in association with EVs and soluble proteins

To better understand the secretion and extracellular features of AAV5-delivered engineered miRNAs, it is important to select an isolation method that achieves higher purification of EVs and separation from protein complexes than precipitating agents. Due to size difference between these particles, efficient separation of EVs from plasma proteins have been achieved by SEC columns in numerous studies (Fig. 4A).[26] Sepharose columns allow for recovery of intact functional vesicles from soluble protein contaminants. In this study, culture media collected from cells transduced with the highest dose of AAV5-miHTT and AAV5-miATXN3 (MOI $1x10^7$) was separated in 26 fractions (n=3 per condition). After separation, fractions were concentrated by centrifugation filters, followed by RNA isolation. The abundance of mature miHTT and miATXN3 molecules was measured in fractions 2 to 21. Both engineered miRNAs were enriched in the SEC fractions corresponding to EVs (fractions 6-8), as well as in the fractions containing the bulk of proteins, protein complexes, and EVs with a diameter < 70 nm (fractions 14-17; Fig. 4B and 4C). Interestingly, endogenous let-7a miRNA was comparably detected in EV- and protein-containing fractions, as opposed to endogenous miR-16, which was only detected in association with protein particles and not within EVs (Supplementary Figure 3). Clean separation of EVs from protein complexes was confirmed by flow cytometry analysis with CD63, CD81 and lactadherin markers. Positive events for all markers were present in a representative EV-containing fraction (fraction 7, Supplementary Figure 3), but significantly less to non-detectable in a protein-containing fraction (fraction 15, Supplementary Figure 3). TEM analysis was used to further visualize and confirm the presence of EVs in fraction 7, as compared to the high amount of protein complexes and absence of EVs in fraction 15 (Fig. 4D). Altogether, these results confirmed the secretion of engineered miRNAs in association with EVs, which protect them from RNases, as well as with soluble proteins. That being the case, the presence and detection of such secreted therapeutics in biological fluids in vivo could be used as translational pharmacokinetics markers for gene therapy.

## Widespread distribution of therapeutic miHTT in non-human primates after intrastriatal treatment with AAV5-miHTT

One of the advantages of miRNA-based lowering gene therapies is the long-term expression after a one-time administration of AAV5 treatment. Monitoring the expression of the active miRNA molecules in accessible biological fluids would add value to dosing, safety and efficacy evaluation in the clinic. In this study, the secretion and successful detection of engineered miRNAs in association within EVs offers the posibility to develop an accessible pharmacokinetic measure for gene therapies injected in the brain. To investigate this, a large animal model is necessary for the appropriate translation of AAV5-miRNA therapeutic spread in a human-like brain size, correspondence to biomarker measures in biofluids, and sampling of sufficient CSF volume to perform these measurements. Therefore, we next assessed the detection and endurance of engineered miRNA molecules in the CSF in non-human primates after one-time intrastriatal administration. The therapeutic candidate for HD, AAV5-miHTT, was intracranially and bilaterally injected in the caudate and putamen, the most affected structures in HD patients (Fig. 5A). Administration of AAV5-miHTT was performed by guided real-time MRI, which mimics the surgical approach and target region planned in ongoing clinical trial. Animals were injected with AAV5-miHTT at two doses (low and high) as indicated in Figure 5B and sacrificed at 6 month (cohort 1) or 24 months (cohort 2).

First, we evaluated the biodistribution and long-term expression of miHTT at six months, and at 24 months after treatment by collection of brain punches across several structures of interest. As expected, the highest level of vector DNA, as well as miHTT molecules were observed in injected areas such as caudate and putamen (striatum) (Supplementary Figure 4 and Fig. 5C). High levels of viral DNA and miHTT expression were also detected in other deep brain structures, including thalamus and midbrain, and in cortical areas from frontal to occipital lobes (Supplementary Figure 4 and Fig. 5C). Comparable levels of miHTT expression were found between the two cohorts at 6 months ant 24 months. Due to a mismatch between the engineered miHTT and the *HTT* primate sequence, the lowering efficacy could not be evaluated in these areas. Low to undetectable levels of miHTT molecules were found in peripheral organs including kidney, liver and spleen (Fig. 5C). This treatment approach shows that a one-time local infusion of AAV5-miHTT is sufficient for widespread and enduring expression of therapeutic miRNAs in the brain of large animals.

## Detection of EV-associated miHTT molecules in CSF from non-human primates after intrastriatal treatment with AAV5-miHTT

To validate the potential use of circulating engineered miRNAs as pharmacokinetic markers of AAV5-miRNA gene therapies directly administered into the CNS, we quantified the presence of EV-associated therapeutic miHTT in biological fluids from non-human https://mc.manuscriptcentral.com/braincom primates after direct brain infusion of AAV5-miHTT. CSF was collected by lumbar puncture from each animal pre-dose and at months 1, 3, 4.5 and 6 (cohort 1) and 12, 15, 18, 21 and 24 (cohort 2) post-treatment (Fig. 6A). Total RNA associated with EVs was isolated by a spin column-based method that allows for enrichment of vesicular over non-vesicular RNA,[34] using spiked cel-miR-39 miRNA as RNA isolation control (Supplementary Figure 5). High levels of EV-associated miHTT molecules were detected in CSF at 6 months after intrastriatal treatment in cohort 1, and comparable levels up to 24 months in cohort 2 (Fig. 6B). Levels of EV-associated miHTT molecules were quantified as fold change expression relative to pre-dose or background of the assay. In both cohorts, a comparable and relatively constant miHTT expression in the CSF was measuredduring the 6-month and 24-month observation period. Despite variations, there were no signifixant differences of miHTT levels across tiempoints. In contrast, viral vector levels in CSF and plasma rapidly declined from day 1, to levels around the lower limit of quantification after four weeks (1 month), and undetectable levels at week 12 (3 months) (Fig. 6C and 6D). Separation of CSF EVs by SEC confirmed the presence of low levels of miHTT molecules in association with EVs, as well as in protein-enriched fractions (Supplementary Figure 5). In conclusion, these results confirm the detection of circulating EV-associated therapeutic miRNAs in CSF and its potential utility as translational pharmacokinetic markers to monitor the expression and persistence of CNS delivered AAV-miRNA-based gene therapies injected in CNS.

## Discussion

Gene therapy for CNS disorders has faced a number of translational challenges that hamper the establishment of clinically relevant readouts. At this moment, AAV5-miHTT treatment (also known as AMT-130 by uniQure) is entering the clinic and first patients have been successfully injected (Clinicaltrial.gov, NCT04120493). For this and future trials, sensitive measurements reliably reflecting the long-term expression of the therapeutic molecules in the brain are needed. In this study, we demonstrated that engineered miRNA profiles in CSF are suitable pharmacokinetic measurements of AAV-based miRNA therapies directly infused into the brain. We reported for the first time that two different AAV5-delivered therapeutic miRNAs are secreted from diseased neuronal cells in association with EVs, and that the detection of such circulating miRNAs in biofluids is a sensitive indicator of long-term (up to two years) therapeutic expression in the brain. Despite the low protein content of CSF and more invasive sampling compared to plasma, the rapid interchange of molecules between CSF and brain interstitial fluid though the glymphatic system makes the CSF a valuable biofluid for CNS biomarker discovery.[35] Moreover, since the blood-brain barrier restricts the passage of large and small molecules in the blood, the quantification of therapeutic miRNAs in the CSF seems to be a more appropriate source for pharmacokinetic measurements specific for CNS. Other studies have focused on the characterization of quantifiable treatment-response biomarkers that correlate with neurodegeneration. Prominent examples of such biomarkers are tau protein and neurofilament light chain, which are suitable general markers for a variety of neurodegenerative disorders.[36,37] However, due to slow disease progression of neurodegenerative diseases in general, significant changes are difficult to measure over a short period of time in a clinical trial. Development of specific pharmacokinetic markers, such as detection of circulating engineered miRNAs, offers the possibility to monitor the active molecule over time, as well as to understand the relationship between dose, efficacy, and biomarker response.

EVs provide a protective and enriched source of specific bioactive molecules, including RNA, lipids and proteins.[18] Next generation sequencing analysis of plasma-derived circulating RNAs suggest that miRNAs are the most abundant EV-associated RNAs.[38] Interestingly, the cellular miRNA composition generally differs from the EV-enriched miRNA profile, suggesting that cells may utilize sorting mechanisms to regulate the selective packaging of miRNAs into EVs. For instance, specific sequence motifs, chromosomal location, 3'end post-transcriptional modifications and miRNA:target mRNA ratio have been reported to influence the sorting of miRNAs into EVs.[39–42] Differences in secretion profile have also been associated with Ago2-dependent processing of pre-miRNA backbones.[25] In this study, the pre-miR-451a backbone, which follows a dicer-independent non-canonical processing by Ago2 protein,[27,43] was selected for the design of our engineered therapeutic miRNAs (miHTT and miATXN3). Despite the different sequence content, the insertion of both engineered miRNA sequences in the pre-miR-451a scaffold resulted in similar secretion profile. Our results support the work by Reshke et al.[44] in which different siRNA were efficiently loaded into EVs when integrated in pre-miR-451 scaffold, but not in pre-miR-16. These studies support the advantage of pre-miR-451 based candidates, not only to deliver safe and efficacious gene therapies, but also for pharmacokinetic and translational extracellular measurements.

A major challenge in the EV research field is the large and not-fully characterized vesicle diversity.[45] Due to discrepancies and methodological limitations to isolate pure subtype populations, the term “extracellular vesicles” has been encouraged to generally refer to all cell-derived, non-replicable, secreted membrane structures.[46] This term includes vesicular subtypes with different origins, including endosome-derived exosomes and shedding vesicles released from plasma membrane. In this context, specification and understanding of the strengths and limitations of the selected methods of isolation is crucial for data interpretation.[46,47] In this study, we evaluated the association of therapeutic miRNAs with EVs by using three different methods of isolation. As a first step, a precipitation-based method for tissue culture media was selected for high particle recovery. Cell culture conditions were optimized to assure the isolation of sufficient number of particles for miRNA detection. Characterization of isolated vesicles by imaging and molecular methods showed vesicles of expected size, shape and membrane composition. However, other RNA-containing non-vesicular particles were highly present.[31] To achieve a better purification of EVs from other soluble component, we selected a size-based separation method.[26] Compared to other conventional EV isolation techniques – such as ultracentrifugation and density gradient – SEC has been recommended as a fast and affordable method for good recovery of vesicles larger than 75nm, with almost complete removal of protein contaminants.[48] We found detectable levels of therapeutic miRNAs in EV-enriched fractions, but also in protein-containing fractions. Besides lipoproteins and Ago2 protein complexes, which have been remarkably associated with circulating miRNAs,[49,50] smaller vesicles might also elute in these fractions.[26,51] Lastly, and due to limited sample availability of CSF, we used a spin column-based method for the direct isolation of RNA from EVs in biofluids.[34] By using different methods of isolation, we confirmed the presence of premiR-451 engineered miRNAs within EVs, as well as in association with soluble proteins, in both cultured media and body fluids. These results reflect the importance of purifying and characterizing extracellular miRNA-binding species in order to avoid misinterpretations.

Apart from its value as a source for translational pharmacokinetic measures, the association of therapeutic miRNAs with EVs might have further implications for gene therapies. Vesicular transfer of miRNAs, lipids and misfolded proteins have been described as a novel cell-to-cell communication pathway.[19,52] Endogenous and viral miRNAs associated with EVs were transferred and delivered into recipient cells by fusion with cell membrane, where they were still functional and lowered the expression of target genes upon transfer.[53,54] Besides their potential role as novel delivery systems, we also postulate that EVs might contribute to the spread and amplification of AAV-based gene therapies throughout the brain. In other words, efficacy of miRNA-based gene therapy could be extended beyond the initial viral transduction via EV-dependent transfer. New methods to track the uptake and content delivery are necessary to characterize the contribution of EV internalization.[55] Further implications of EV-associated therapeutic miRNAs for successful gene therapies still need to be investigated.

Altogether, our results show the potential of EV-associated artificial miRNAs in CSF as pharmacokinetic markers for monitoring long-term expression of AAV-miRNA gene therapies directly delivered in the brain. The present findings contribute to the better understanding of the transfer of therapeutic miRNAs between cells and body compartments. This knowledge is highly relevant to optimize delivery, therapeutic distribution, dose translation, transgene persistence and efficacy monitoring in the first clinical trial of miRNA-based gene therapies for brain diseases.

**Acknowledgements:** The authors are grateful to the team of Process Development and Analytical Development at uniQure for the production and characterization of AAV5-miHTT and AAV5-miATXN3. Special thanks should be given to Jacek Lubelski, Erich Ehlert, Tamar Grevelink, Mark van Veen, and Maroeska Oudshoorn. The authors also thank Ellen Broug, Eileen Sawyer and David Lickorish at uniQure for critically reviewing the manuscript. We are also grateful for the research and technical input to Linda G. Rikkert, Najat Hajji and Chi M. Hau from Laboratory of Experimental Clinical Chemistry at Amsterdam UMC, and to Nicole N. van der Wel and Anita E. Grootemaat for Electron Microscopy analysis at Cellular Imaging core facility of the Amsterdam UMC.

**Funding:** This research project was funded by uniQure biopharma B.V.

**Competing interests:** MSG, MME, AV, CVT, JS, AS, SK, EAS, PK, SvD were employees and shareholders at uniQure at the time this woek was conducted. MDH has close affiliations with uniQure. Filed patent applications pertaining to the results presented in this paper include the following: WO2016102664 (resulting in at least US 10,174,321 and EP 3237618B1), WO2020104435A1 and, WO2020104469A1.

## FIGURE LEGENDS

**Fig. 1**. **Design and mechanism of action of AAV5-miRNA gene therapies.** (**A**) Engineered miRNAs in which the guide strand of pre-miR-451a has been modified to specifically target human HTT (miHTT) and ataxin3 (miATXN3) mRNA sequences. (**B**) Schematic of mechanism of action of AAV5-miRNA in neuronal cells. Upon receptor recognition, AAV5 is internalized and transported to the nucleus where the expression cassette remains episomal. The engineered miR-451a is expressed and processed by the endogenous celullar machinery into mature miRNA molecules, which are loaded into the RNA-induced silencing complex. Recognition and binding of the active miRNA to target mutant mRNA results in mRNA degradation and reduction of protein translation. In this study, we investigate the hypothesis that mature miRNA molecules are secreted via exosomes and microvesicles, also known as extracellular vesicles (EV), or associated with lipoproteins and protein complexes (such as AGO2).

**Fig. 2**. **AAV5-delivered therapeutic miRNAs are secreted by iPSC-derived neurons in a dose-dependent manner.** (**A and B)** Dose-dependent transduction of neuronal cells by AAV5-miHTT (A) and AAV5-miATXN3 (B) (n=3 culture plates/group). Results are expressed as AAV5 genome copies/ug DNA (each dot representing independent cell culture and average ± SEM indicated). (**C** and **D**) Dose-dependent expression of miHTT (C) and miATXN3 (D) molecules in neuronal cells (n=3 culture plates/group. (**E** and **F**) Detection of EV-associated miHTT (E) and miATXN3 (F) molecules secreted from neuronal cells at 5 and 12 days after treatment. (**G, H** and **I**) Correlation analysis of AAV5 dose (log10) and relative cellular miHTT expression (G), AAV5 dose (log10) and relative EV-associated miHTT detection (H) , and relative cellular miHTT expression and EV-associated miHTT detection (I) (n=6, each dot represents the average value of an independent experiment in triplicates ). Linear regression anaysis indicating coefficient of determination ($R^2$) and p-value (p).

**Fig. 3. Characterization of EVs isolated from culture media by precipitation.** (**A**) Western-blot analysis of cell lysate (left lane) and EV pellet (right lane) with exosomal markers (CD63, Alix and TSG-101), microvesicles (Calnexin), cytoplasm (a-tubulin), RISC complex (Ago2) and high-density lipoproteins (HDL, ApoE). (**B**) Particle size distribution of vesicles measured by microfluidic resistive pulse sensing (nCS1). (**C**) Representative image of transmission electron microscopy (TEM) analysis of vesicles precipitated from culture media of AAV5-miATXN3 transduced cells (right). Scale bar 200nm. (**D**) Relative levels of miATXN3, miR-16, let-7a and spike-in cel-miR39 in EV pellets after RNase treatment (black bars) and TritonX-100 (detergent) together with RNase (grey bars) compared to non-treated EV pellets isolated by precipitation. Bars represent average ± SEM.

**Fig. 4. Detection of therapeutic miRNAs associated with EVs and protein complexes secreted from AAV5-treated iPSC-derived neurons**. (**A**) Schematic of AAV5-miRNA transduction of iPS-derived neurons and isolation of EVs and protein complexes by size exclusion chromatography (SEC). (**B** and **C**) Detection of miHTT (B) and miATXN3 (C) molecules in different SEC fractions corresponding to EV- (blue) and protein- (orange) containing fractions complexes. (**D**) Flow cytometry analysis of fraction 7 (EV fraction), showing positive signal for CD63, CD81 and lactadherin markers, and of fraction 15 (soluble protein), showing low or no detection signal for CD63, CD81 and lactadherin. (**E**) Representative images of TEM analysis of vesicles isolated by SEC in fraction 7 (left) and abundant proteins in fraction 15 (right). Scale bar 200nm.

**Fig. 5. Widespread distribution and expression of miHTT in non-human primates after intrastriatal guided injection.** (**A**) Non-human primate coronal brain section indicating the placement of bilateral injection in caudate and putamen by MRI-guided convention-enhanced delivery (CED). (**B**) Study design and treatment groups with different doses of AAV5-miHTT. (**C**) Biodistribution of miHTT expression in different brain areas. Scheme on the left indicates color-coded brain regions, corresponding to the colors on the right graph. Bars represent average ± SEM of miHTT (molecules/ µg input RNA).

**Fig. 6. Detection of therapeutic miHTT in CSF at6 months and 24 months after local brain administration in non-human primates**. (**A**) Scheme of local intrastriatal injection of AAV5-miHTT in non-human primates and longitudinal collection of CSF by lumbar puncture at different time points for cohort 1 and cohort 2. (**B**) Fold change expression of EV-associated miHTT molecules in the CSF detected by TaqMan qPCR at different time points after striatal treatment at 6 months in cohort 1 (low dose and high dose, n=6 per dose group) and at 24 months in cohort 2 (high dose, n=4) (average ± SEM per time point are indicated). (**C** and **D**) Quantification of vector DNA in plasma (C) and CSF (D) at different time points after striatal treatment in cohort 1 with low and high doses of AAV5-miHTT. LOD: Limit of detection.

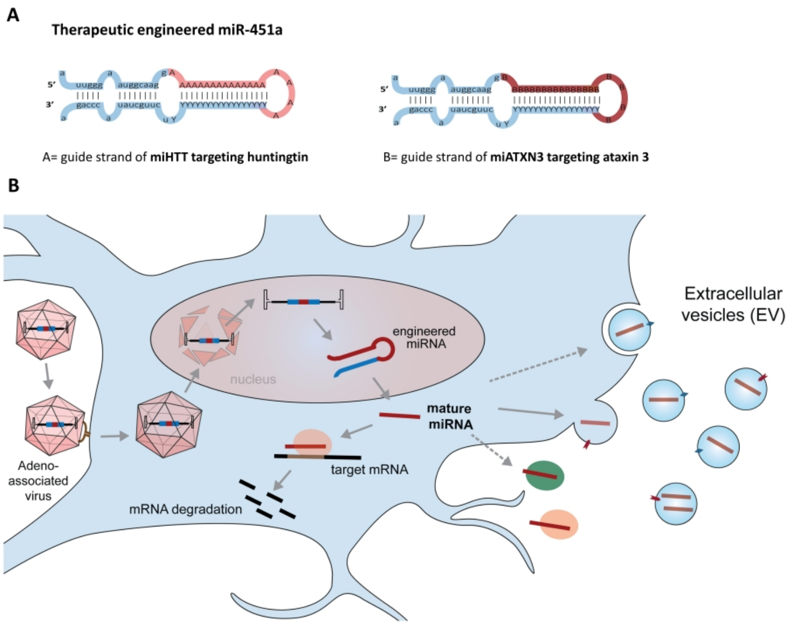

Fig. 1. Design and mechanism of action of AAV5-miRNA gene therapies. (A) Engineered miRNAs in which the guide strand of pre-miR-451a has been modified to specifically target human HTT (miHTT) and ataxin3 (miATXN3) mRNA sequences. (B) Schematic of mechanism of action of AAV5-miRNA in neuronal cells. Upon receptor recognition, AAV5 is internalized and transported to the nucleus where the expression cassette remains episomal. The engineered miR-451a is expressed and processed by the endogenous celullar machinery into mature miRNA molecules, which are loaded into the RNA-induced silencing complex. Recognition and binding of the active miRNA to target mutant mRNA results in mRNA degradation and reduction of protein translation. In this study, we investigate the hypothesis that mature miRNA molecules are secreted via exosomes and microvesicles, also known as extracellular vesicles (EV), or associated with lipoproteins and protein complexes (such as Ago2).

599x450mm (300 x 300 DPI)

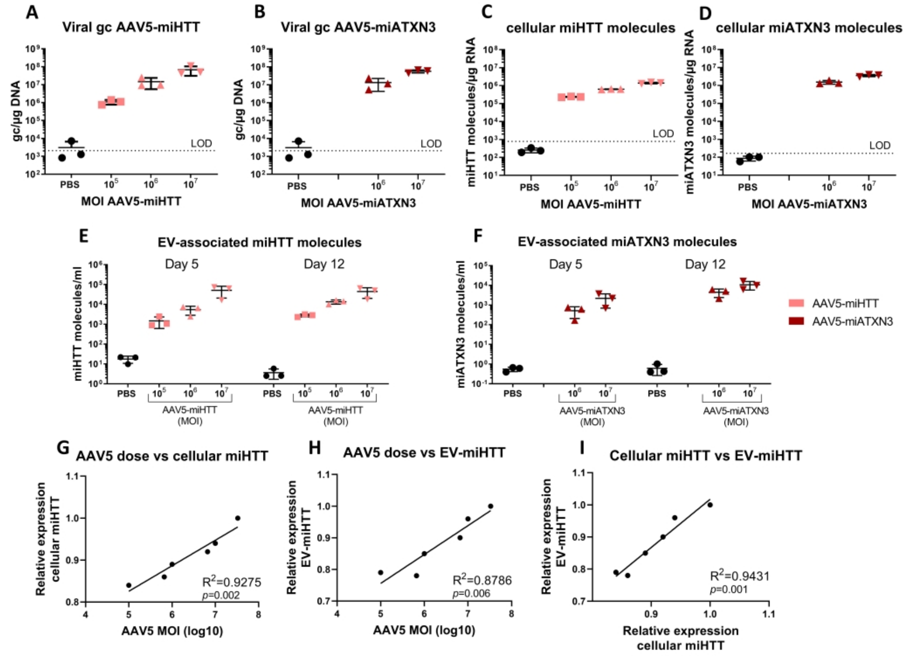

Fig. 2. AAV5-delivered therapeutic miRNAs are secreted by iPSC-derived neurons in a dose-dependent manner. (A and B) Dose-dependent transduction of neuronal cells by AAV5-miHTT (A) and AAV5-miATXN3 (B) (n=3 culture plates/group). Results are expressed as AAV5 genome copies/µg DNA (each dot representing independent cell culture and average ± SEM indicated). (C and D) Dose-dependent expression of miHTT (C) and miATXN3 (D) molecules in neuronal cells (n=3 culture plates/group. (E and F) Detection of EV-associated miHTT (E) and miATXN3 (F) molecules secreted from neuronal cells at 5 and 12 days after treatment. (G, H and I) Correlation analysis of AAV5 dose (log10) and relative cellular miHTT expression (G), AAV5 dose (log10) and relative EV-associated miHTT detection (H) , and relative cellular miHTT expression and EV-associated miHTT detection (I) (n=6, each dot represents the average value of an independent experiment in triplicates ). Linear regression analysis indicating coefficient of determination (R2) and p-value (p).

599x450mm (300 x 300 DPI)

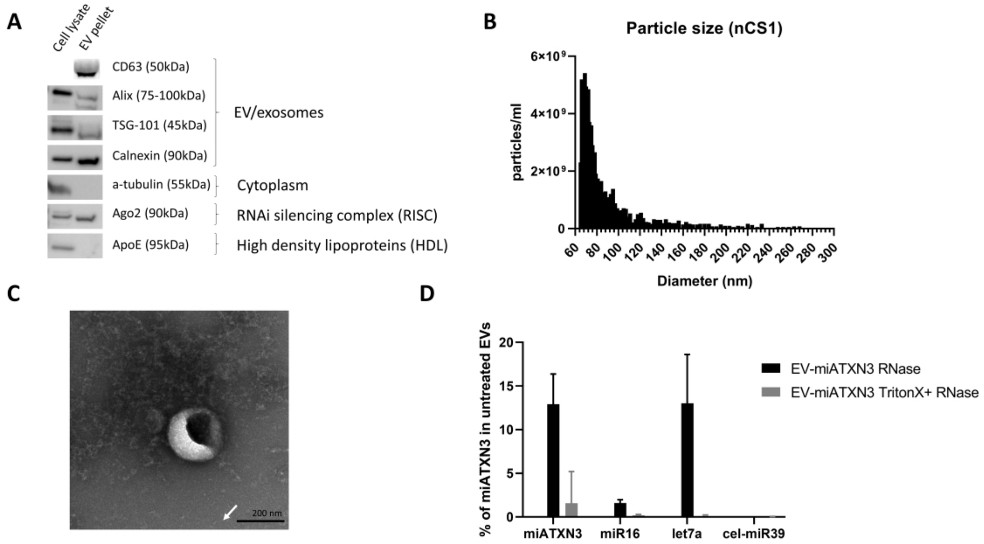

Fig. 3. Characterization of EVs isolated from culture media by precipitation. (A) Western-blot analysis of cell lysate (left lane) and EV pellet (right lane) with exosomal markers (CD63, Alix and TSG-101), microvesicles (Calnexin), cytoplasm (a-tubulin), RISC complex (Ago2) and HDL (ApoE). (B) Particle size distribution of vesicles measured by microfluidic resistive pulse sensing (MRPS nCS1). (C) Representative image of TEM analysis of vesicles precipitated from culture media of AAV5-miATXN3 transduced cells (right). Scale bar 200nm. (D) Relative levels of miATXN3, miR-16, let-7a and spike-in cel-miR39 in EV pellets after RNase treatment (black bars) and TritonX-100 (detergent) together with RNase (grey bars) compared to non-treated EV pellets isolated by precipitation. Bars represent average ± SEM.

500x299mm (300 x 300 DPI)

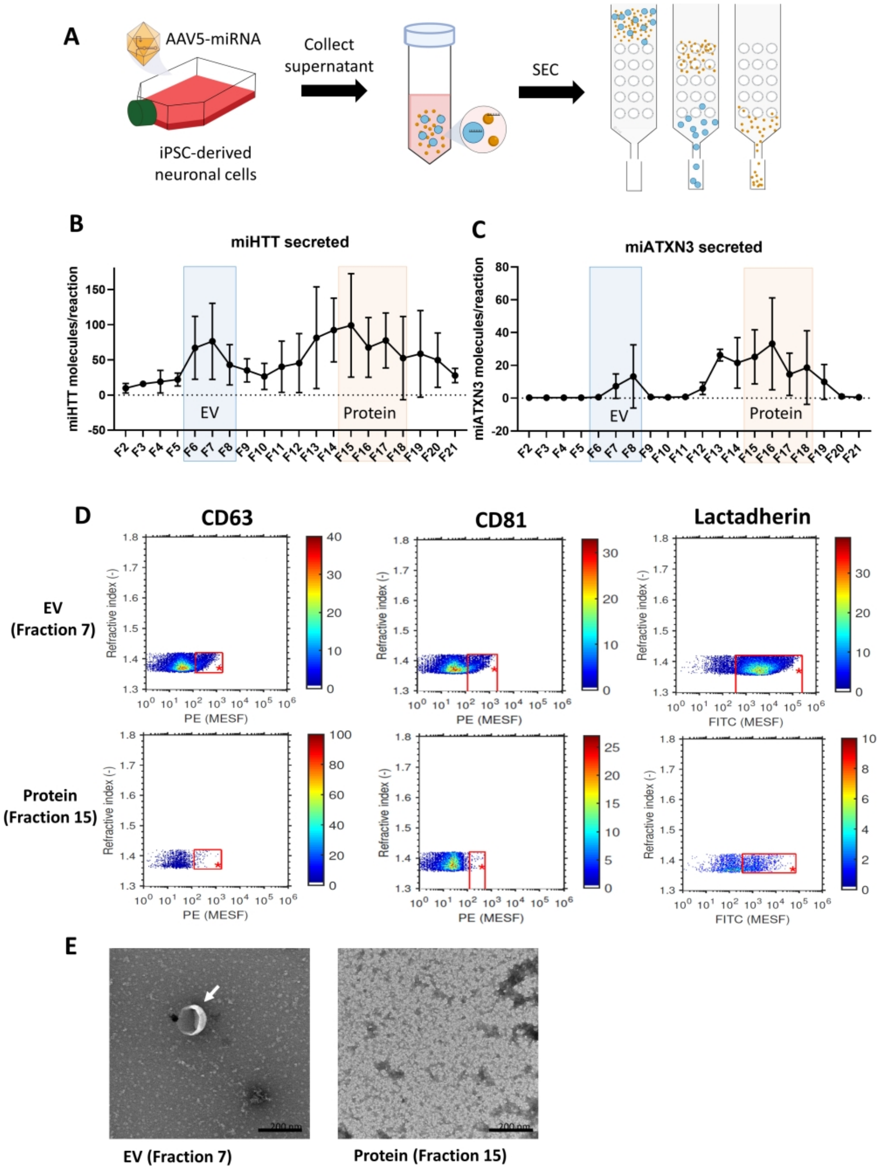

Fig. 4. Detection of therapeutic miRNAs associated with EVs and protein complexes secreted from AAV5-treated iPSC-derived neurons. (A) Schematic of AAV5-miRNA transduction of iPSC-derived neurons and isolation of EVs and protein complexes by SEC (B and C) Detection of miHTT (B) and miATXN3 (C) molecules in different SEC fractions corresponding to EV- (blue) and protein- (orange) containing fractions complexes. (D) Flow cytometry analysis of fraction 7 (EV fraction), showing positive signal for CD63, CD81 and lactadherin markers, and of fraction 15 (soluble protein), showing low or no detection signal for CD63, CD81 and lactadherin. (E) Representative images of TEM analysis of vesicles isolated by SEC in fraction 7 (left) and abundant proteins in fraction 15 (right). Scale bar 200nm.

299x350mm (300 x 300 DPI)

A

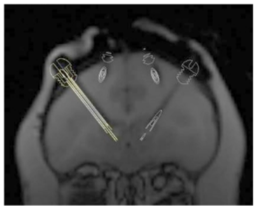

B

| | Treatment AAV5-miHTT | Dose level (gc/brain) and volume (ul) | | Number of animals | |
|---|---|---|---|---|---|
| | | Dose level | Volume | Male | Female |
| **Cohort 1** (6 months) | Low dose | $2 \times 10^{12}$ | 4 x 100 | 3 | 3 |
| | High dose | $2 \times 10^{13}$ | 4 x 100 | 3 | 3 |
| **Cohort 2** (24 months) | High dose | $2 \times 10^{13}$ | 4 x 100 | 2 | 2 |

C

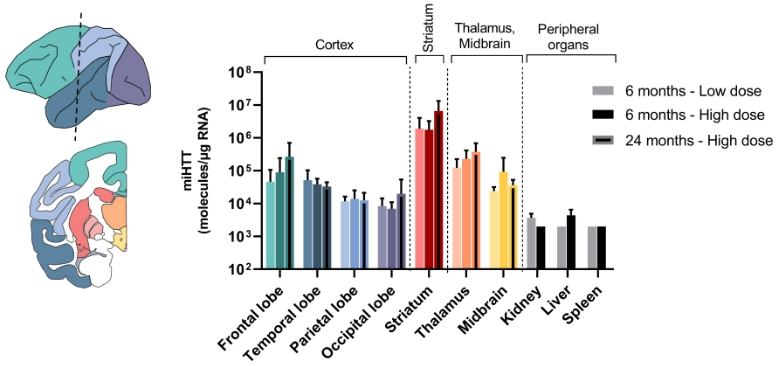

Fig. 5. Widespread distribution and expression of miHTT in non-human primates after intrastriatal guided injection. (A) Non-human primate coronal brain section indicating the placement of bilateral injection in caudate and putamen by MRI-guided CED. (B) Study design and treatment groups with different doses of AAV5-miHTT. (C) Biodistribution of miHTT expression in different brain areas. Scheme on the left indicates color-coded brain regions, corresponding to the colors on the right graph. Bars represent average ± SEM of miHTT (molecules/μg input RNA).

559x397mm (300 x 300 DPI)

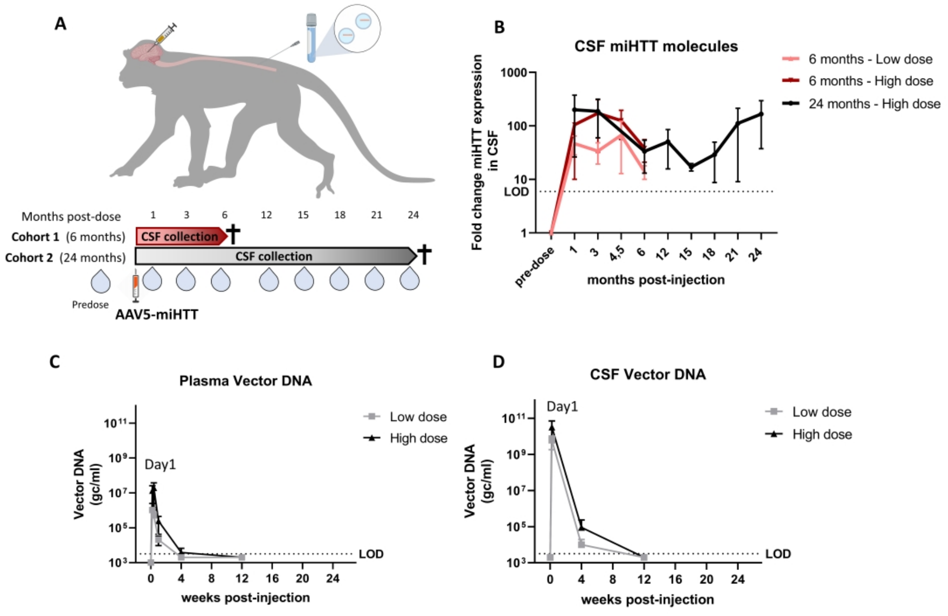

Fig. 6. Detection of therapeutic miHTT in CSF at 6 months and 24 months after local brain administration in non-human primates. (A) Scheme of local intrastriatal injection of AAV5-miHTT in non-human primates and longitudinal collection of CSF by lumbar puncture at different time points for cohort 1 and cohort 2. (B) Fold change expression of EV-associated miHTT molecules in the CSF detected by TaqMan qPCR at different time points after striatal treatment up to 6 months in cohort 1 (low dose and high dose, ns=6 per dose group) and 24-months in cohort 2 (high dose, ns=4) (average ± SEM per time point are indicated). (C and D) Quantification of vector DNA in plasma (C) and CSF (D) at different time points after striatal treatment in cohort 1 with low and high doses of AAV5-miHTT. LOD: Limit of detection.

559x397mm (300 x 300 DPI)

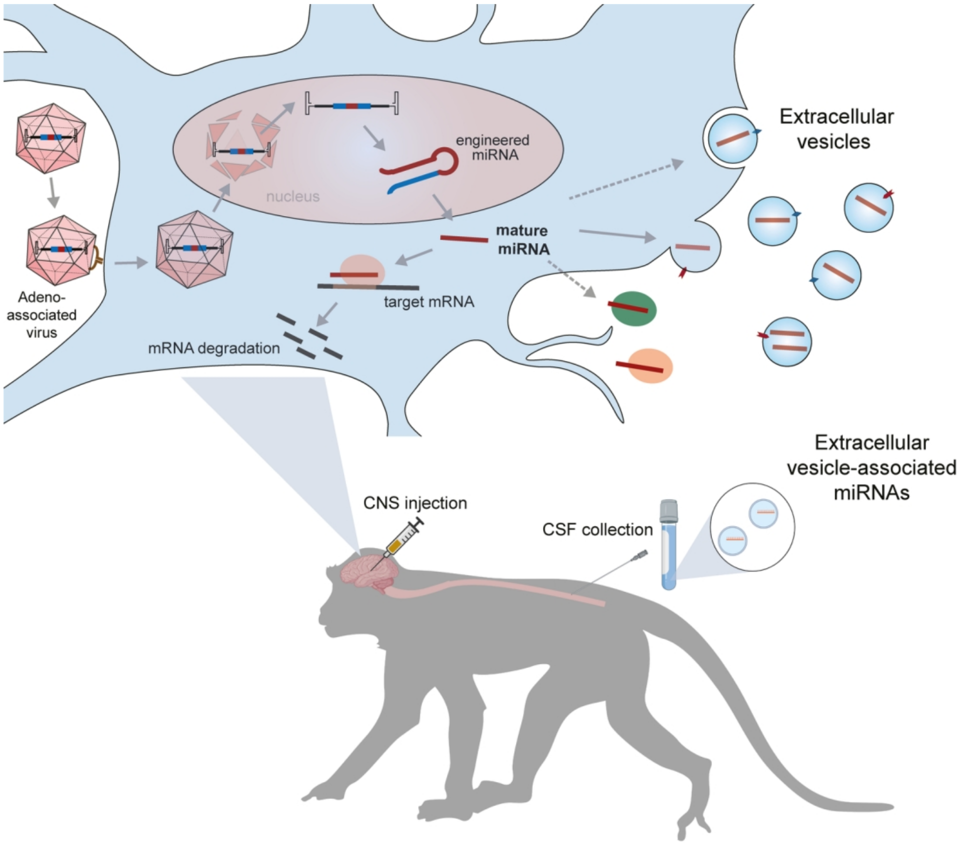

Graphical Abstract

143x125mm (300 x 300 DPI)

**Abbreviated summary**

Sogorb-Gonzalez et al. report that adeno-associated viral vector-delivered therapeutic microRNAs are secreted from neuronal cells within extracellular vesicles, and that circulating extracellular vesicle-associated microRNAs in CSF can be used to monitor the durability of microRNA-based gene therapies for CNS disorders up to two years after one time brain infusion.

## Supplementary Material

fcab054_Supplementary_Data

