# Supplementary material for: Secreted therapeutics: monitoring durability of microRNA-based gene therapies in the central nervous system
Source: Brain Commun. 2021 Apr 1;3(2):fcab054. doi: 10.1093/braincomms/fcab054 (PMC8093922; doi:10.1093/braincomms/fcab054)
Supplement: fcab054_Supplementary_Data [file fcab054_supplementary_data.docx]

# Supplementary Figures


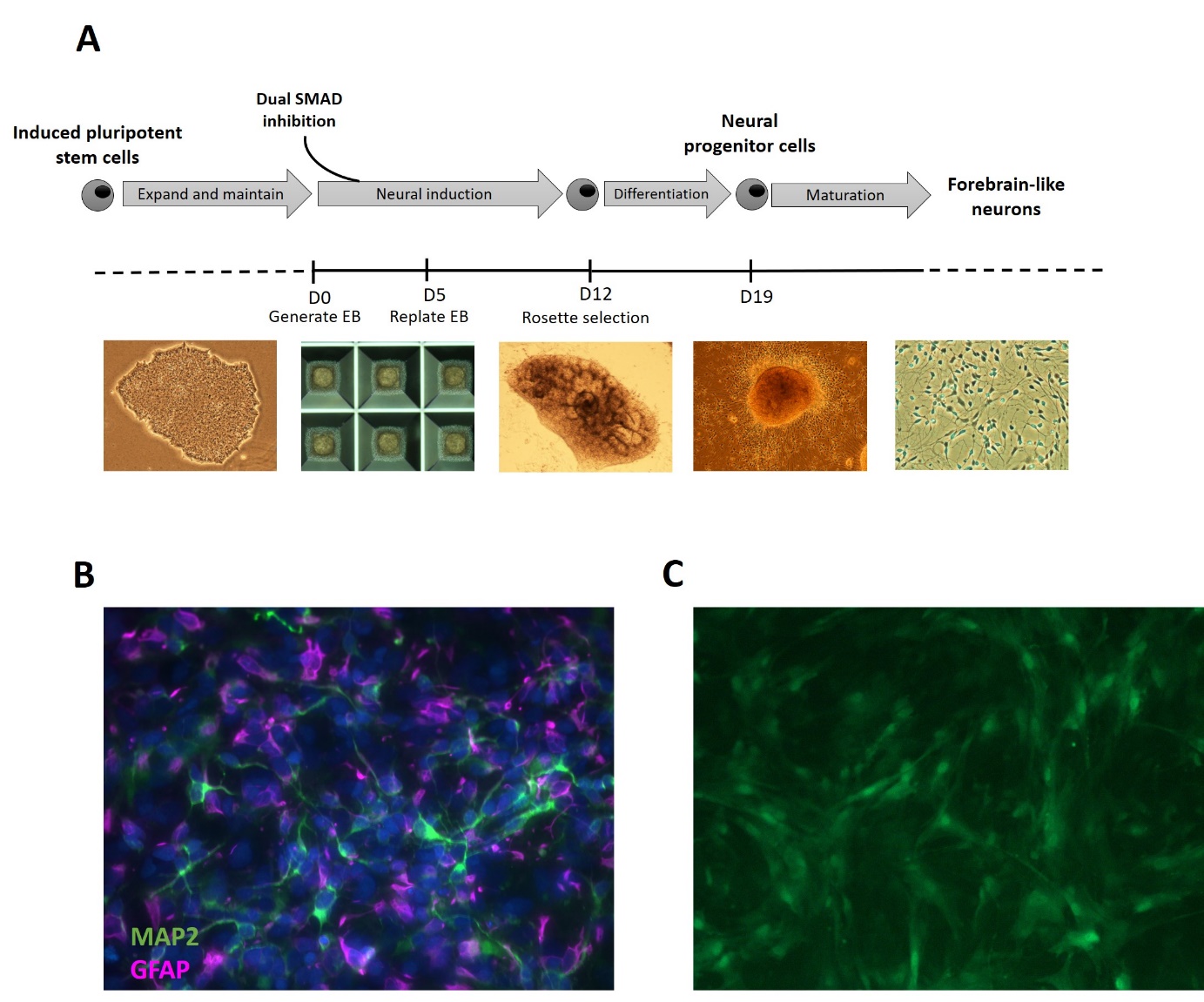


**Supplementary Figure 1.** (**A**) Differentiation of induced pluripotent stem cells to forebrain-like neurons. (**B**) Immunocytochemistry of 2-week matured neuronal cultures positive for neuronal (MAP2, green) and astrocytic (GFAP, magenta) markers. DAPI (blue) represents the nucleus of the cells. (**C**) Transduction of neuronal cultures with AAV5-GFP (high dose). Representative picture of GFP expression at day 5.


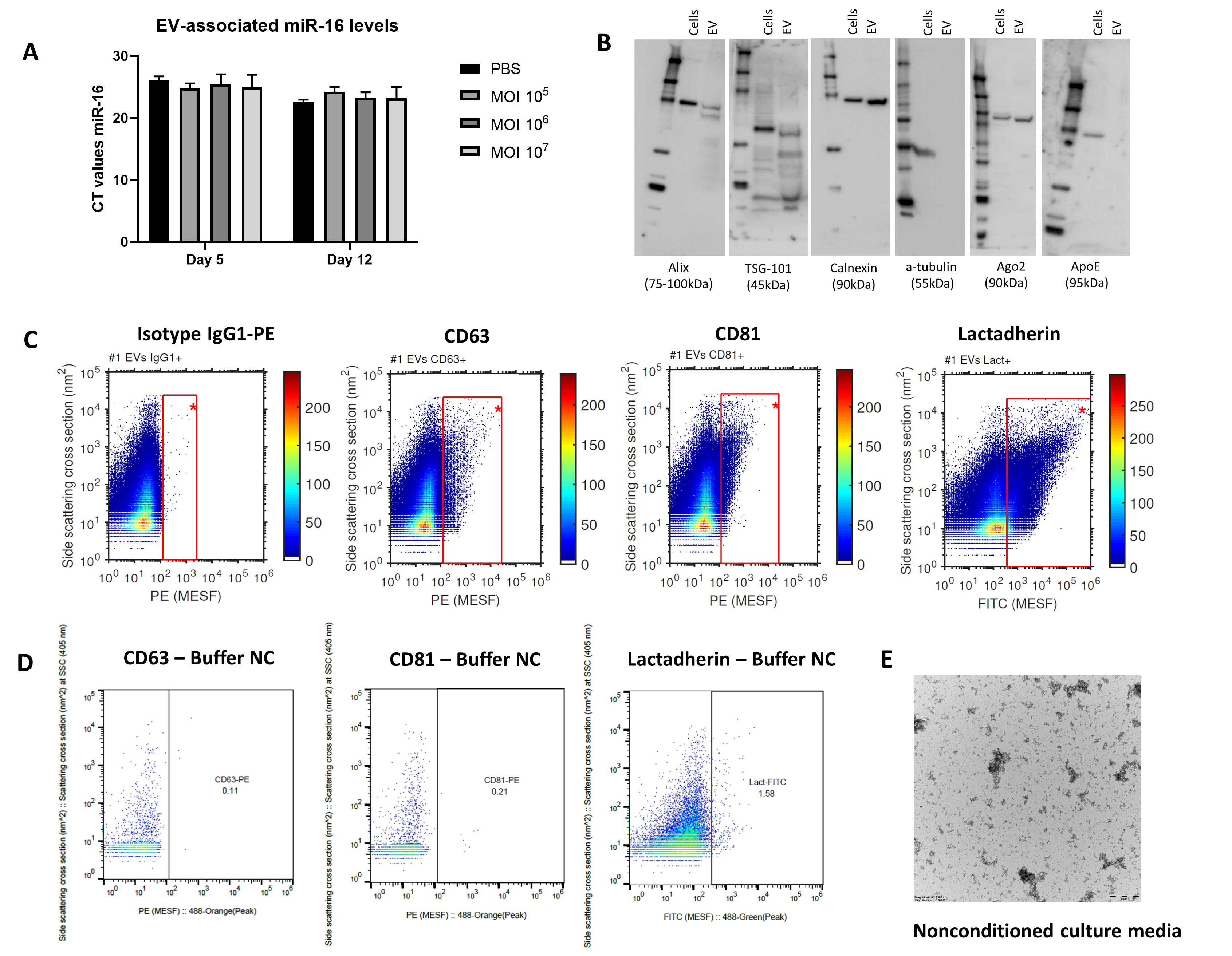


**Supplementary Figure 2**. (**A**) Quantification of endogenous miR-16 levels in EV pellets isolated by precipitation from culture media of neuronal cells at day 5 and 12. (B) Uncropped western blots from for EV and cell markers from Figure 3. (**C**) Flow cytometry results raw data. (**D**) Flow cytometry results of buffers as negative controls (NC) for markers CD63, CD81 and lactadherin. (**E**) TEM picture of particles isolated from non-conditioned culture media


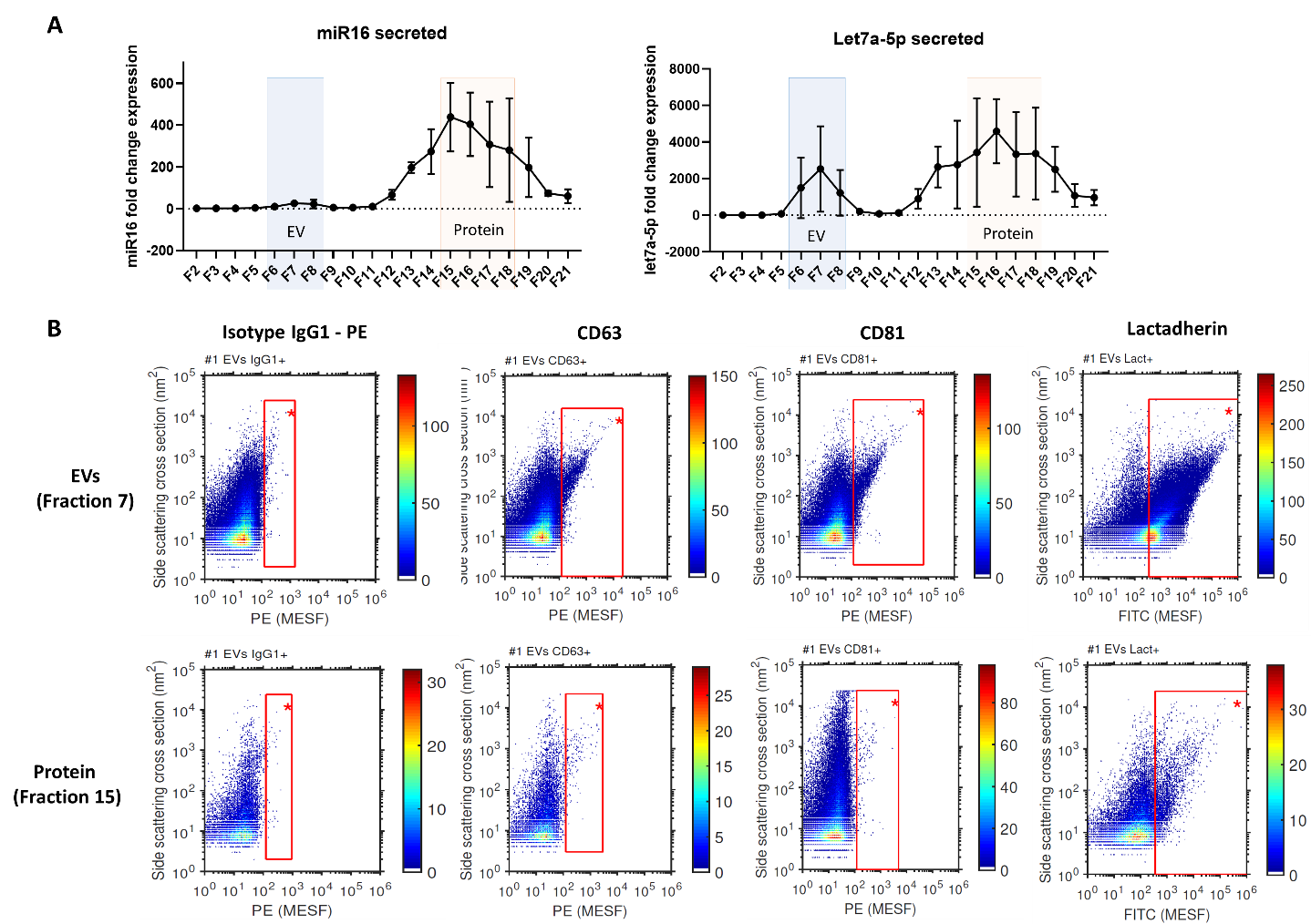


**Supplementary Figure 3**. (**A**) Quantification of secreted endogenous miR-16 (left) and Let7a-5p (right) miRNAs by TaqMan qPCR in fractions 2-21 separated by SEC from culture media of neuronal cells. (**B**) Flow cytometry results raw data of a representative EV fraction (fraction 7) and protein fraction (fraction 15).


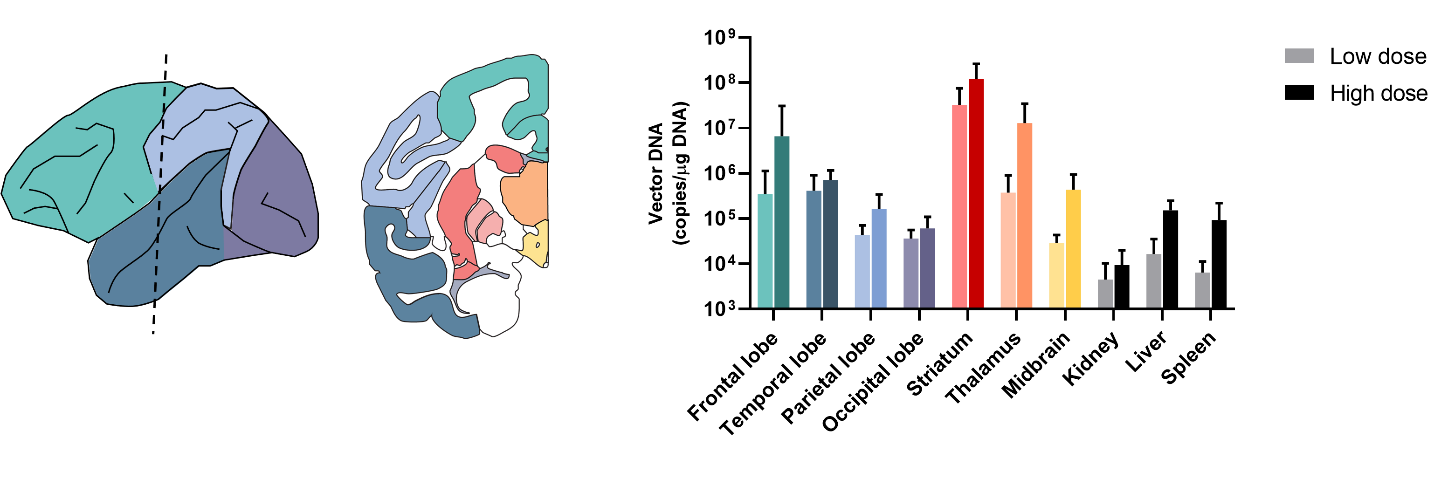


**Supplementary Figure 4.**  Transduction of different brain areas of cohort 1 (6 months) represented by vector DNA (copies/μg DNA) after intrastriatal injection of AAV5-miHTT (low and high dose). Scheme on the left indicates color-coded brain regions, corresponding to the colors on the right graph. Bars represent average ± SEM of miHTT (molecules/ µg input RNA).


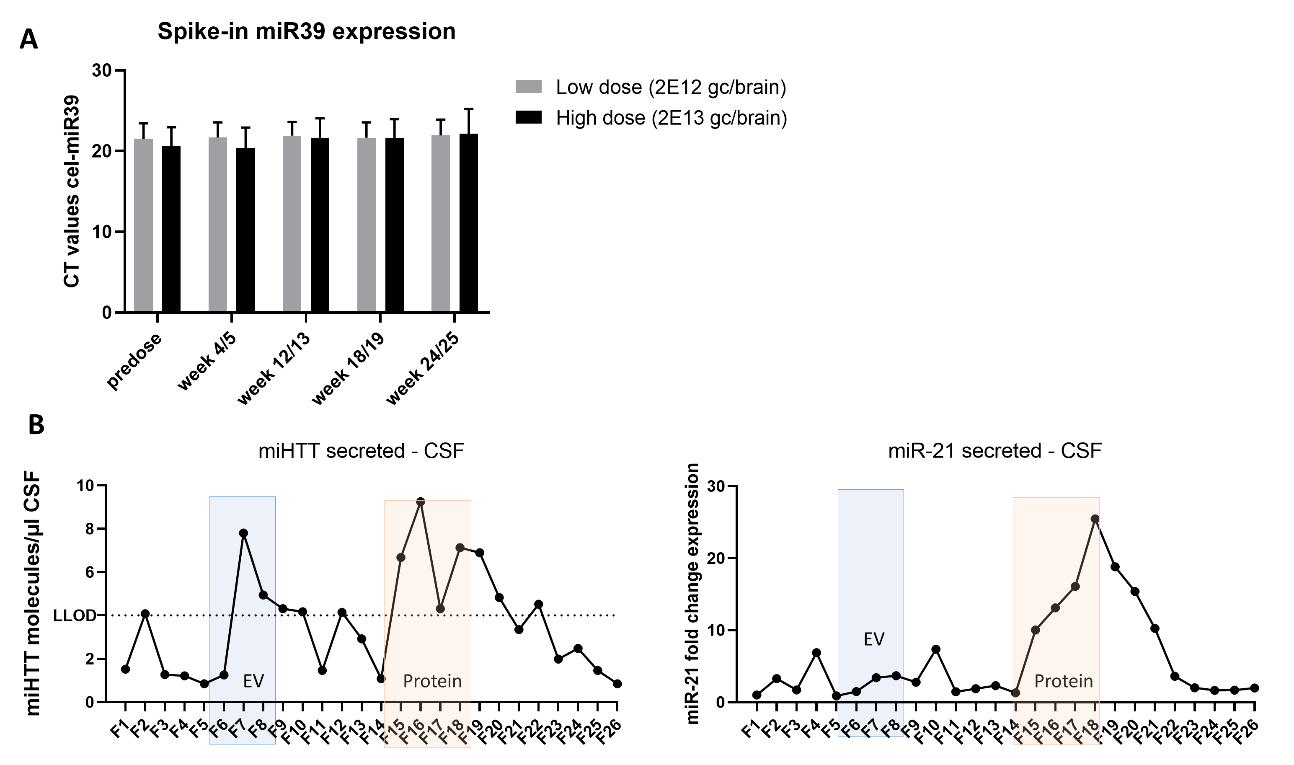


**Supplementary Figure 5.** (**A**) Quantification of spike-in cel-miR-39 by TaqMan qPCR (CT values) after EV-associated RNA isolation from CSF samples. (**B**) Quantification of therapeutic miHTT (left) and endogenous miR-21 (right) in fractions 1-26 separated by SEC from CSF samples.
